# Audience response technology: Engaging and empowering non-medical prescribing students in pharmacology learning

**DOI:** 10.1186/1472-6920-10-73

**Published:** 2010-10-27

**Authors:** Joanne S Lymn, Alison Mostyn

**Affiliations:** 1 School of Nursing, Midwifery & Physiotherapy, University of Nottingham, Queens Medical Centre, Nottingham, UK; 2 School of Veterinary Medicine and Science, University of Nottingham, Sutton Bonington Campus, Loughborough, UK

## Abstract

**Background:**

Non-medical prescribing (NMP) is a six month course for nurses and certain allied health professionals. It is critical that these students develop a good understanding of pharmacology; however, many students are mature learners with little or no formal biological science knowledge and struggle with the pharmacology component. The implications for patient safety are profound, therefore we encourage students not just to memorise enough pharmacology to pass the exam but to be able to integrate it into clinical practice. Audience response technology (ART), such as the KeePad system (KS) has been shown to promote an active approach to learning and provide instant formative feedback. The aim of this project, therefore, was to incorporate and evaluate the use the KS in promoting pharmacology understanding in NMP students.

**Methods:**

Questions were incorporated into eight pharmacology lectures, comprising a mix of basic and clinical pharmacology, using TurningPoint software. Student (n = 33) responses to questions were recorded using the KS software and the percentage of students getting the question incorrect and correct was made immediately available in the lecture in graphical form. Survey data collected from these students investigated student perceptions on the use of the system generally and specifically as a learning tool. More in depth discussion of the usefulness of the KS was derived from a focus group comprising 5 students.

**Results:**

100% of students enjoyed using the KS and felt it promoted their understanding of key concepts; 92% stated that it helped identify their learning needs and 87% agreed that the technology was useful in promoting integration of concepts. The most prevalent theme within feedback was that of identifying their own learning needs. Analysis of data from the focus group generated similar themes, with the addition of improving teaching. Repeated questioning produced a significant increase (p < 0.05) in student knowledge of specific pharmacological concepts.

**Conclusions:**

The use of ART enhanced non-medical prescribing students' experience of pharmacology teaching. Student perceptions were that this system increased their ability to identify learning needs and promoted understanding and integration of concepts. Students also reported that the technology aided exam revision and reduced associated anxiety.

## Background

In the U.K., the government drive to improve patients access to medicines has resulted in an expansion of prescribing rights to a range of non-medical professionals including nurses, pharmacists, physiotherapists, radiographers and podiatrists [1,2]. Indeed on successful completion of a non-medical prescribing (NMP) course at an accredited Higher Education Institute (HEI), these health professionals have access to almost the same formulary of drugs as doctors. The NMP course is delivered part-time over a six month period and consists of 26 taught days within the HEI and 12 days of supervised clinical practice. The taught days comprise a mixture of lectures, tutorials and practical sessions covering the legal, professional and ethical issues in relation to drug prescribing alongside both fundamental and applied pharmacology and consultation skills. There are seven separate assessments which must all be passed to successfully qualify as a non-medical prescriber.

Pharmacology is the biggest single component of the NMP course, and in terms of patient safety is probably the most important, but is the aspect that students struggle with most. This is particularly true given that our recent data has shown that the students who attend this course are mature learners (frequently over 40 years of age), from diverse academic backgrounds, many of whom have little or no formal biological science knowledge [3]. Evidence suggests that students avoid biological sciences, including pharmacology, as it is a learning area perceived as more "difficult" than other aspects of the undergraduate curriculum [4]. This perception is mirrored by lecturers and is perhaps the reason why pharmacology has been neglected in undergraduate teaching, leading to a low level of understanding in qualified nurses [4-6]. The implications of poor pharmacology knowledge for safe and effective prescribing are profound and have been recognised by both the nursing and medical professions [7-9]. The aim of the NMP course must therefore be to encourage students not just to memorise enough pharmacology to pass the exam, but to assimilate this knowledge and be able to integrate it into clinical practice.

While a number of new technologies, including reusable learning objects (RLOs), which are short, interactive tutorials on defined topics [3], and podcasts, have been utilised to enhance student understanding of pharmacology [3,10] these methods are limited by issues such as cost, student familiarity with computers and technology and home access to technology. Moreover these technologies do not provide instant formative feedback for students or lecturers, and misunderstandings of key concepts are often not detected until the summative exam.

Audience response technology, comprises of several small handheld devices and a wireless receiver connected to the computer delivering the lecture. The handheld devices vary from system to system, but usually resemble a small remote control with several keypads labelled 1-10 or A-F and sometimes "true" and "false" keypads; similar to those used for "ask the audience" questions in the television programme "Who Wants to Be a Millionaire?". The handheld devices are distributed to class members who respond to questions delivered during the lecture, the wireless receiver collates the answers and a results graph is produced. More comprehensive information relating to the different systems available can be found in a recently published review [11].

A comprehensive review of the use of ART in higher education has recently been published by Kay and LeSage (2009) who outline the lack of peer-reviewed articles on the use of ART, despite the publication of several review articles [12]. The use of audience response technology, to promote effective student learning is however supported by recent pedagogical research. As reviewed by Jones et al, the use of audience response technology adheres to many of Chickering and Gamson's principles of education [13]; in particular, the active approach to learning, prompt formative feedback, diverse learning styles, increased interaction and opportunities for reflection on knowledge [14]. All of these aspects have been shown to increase both information retention and to promote 'deeper' approaches to learning [15-17]. The use of audience response technology within a lecture promotes active learning, by engaging students with the learning process [18]. Indeed in recent literature students have reported that ART is easy to use [19] and promotes participation and attention in class [19-21]. While these reports are from a variety of student types and across a number of subjects it is worth noting that they have all been conducted outside the UK with most studies being performed in the USA [15,16,19-21]. There are currently no data regarding the use of ART in the UK higher education system in general let alone its use in non-medical prescribing.

For NMP students solving problems posed by the audience response technology involves reading the slides, writing notes and discussing the topic, thus engaging the students in tasks such as analysis, synthesis, and evaluation [15-17]. Prompt, individual feedback is essential to developing understanding in students who have arrived at university through less conventional routes, and who may not have the confidence to ask questions in the lecture environment or to approach the lecturer directly [22,23]. The type of feedback provided by the KS could be described as direct, correct response feedback; informing the learner of the correct answer to a specific problem [24], and as a consequence, areas of learning need. As reviewed by Black and Williams, formative feedback is known to improve student learning [25]. Early formative feedback also provides information to teaching staff about the areas in which students have developed expertise and the teachers can tailor their teaching to address any problems before they can impact on future sessions [26]. Participation in formative assessment has previously been demonstrated to be a predictor of success in summative assessments in health science students [27] and we confirm that this is the case with the NMP course through correlation of formative and summative results from the previous 3 cohorts (R^2 ^= 0.49, P < 0.001 (Spearman's rank test)). The provision of early formative feedback also allows students to reflect upon their knowledge and identify their own learning needs, thus promoting self-directed study and independence.

There are a number of models which outline learning styles, the VARK model proposes four learning styles, visual, auditory, reading/writing and kinaesthetic as well as multi-modal learners who utilise a combination of all four styles. The learning styles of students attending the NMP course will be varied, but our use of multi-media teaching styles incorporating podcasts (auditory), RLOs (auditory and visual) and use of the KS will incorporate all four styles. Evidence suggests that students do prefer multiple learning styles. Audience response technology has been used successfully in other University of Nottingham departments, including Veterinary Medicine who have demonstrated that multiple teaching methods are successful across a range of learning styles and academic backgrounds [28].

Increased interactivity or participation is one of the most regularly cited reasons for integrating audience response technology into teaching [12]. The introduction of audience response technology is useful in encouraging students who would be reluctant to raise their hand to engage in the debate or question session anonymously without fear of humiliation at answering a question incorrectly and also allowing complete honesty, which a show of hands would not provide.

One particular pedagogical area which may be impacted upon by the use of audience response technology is that of threshold concepts. Threshold concepts are described by Meyer and Land as being akin to a portal, representing "a transformed way of understanding, or interpreting, or viewing something without which the learner cannot progress" [29]. For example, threshold concepts such the key pharmacological terms "agonist" and "antagonist" are introduced early in the module; if students do not understand these concepts, they will struggle with the clinical application of these concepts later in the module [29]. By reinforcing the importance of key concepts through regular KS questions, there is ample opportunity for students to ensure that they have made the transformative step in understanding the key concepts.

Additionally, the early formative feedback will allow teaching staff to detect areas of student weakness immediately and thus allow these problem areas to be addressed in more detail within the appropriate session before they can impact on future sessions. The NMP team at the University of Nottingham already provide a number of supportive learning tools for this course, including the use of RLOs and podcasts. It is hoped that the audience response technology will add to, and enhance, this variety of learning & teaching methods by encouraging integration of pharmacological knowledge across different sessions which is critical for ensuring safe and effective prescribing for patients.

The aim of this study was to investigate the use of audience response technology, specifically the KS, to engage NMP students in pharmacology teaching.

## Methods

### Participants

All students attending the non-medical prescribing course at the University of Nottingham between January 2009 and July 2009 (n = 33) were part of this study. As an evaluation of a new teaching methodology this study did not require ethical approval. The study was approved by the Centre for Integrative Learning (University of Nottingham) and the experimental design and analysis was performed following the British Educational Research Association's code of ethics (2004). All data was annonymised before publication.

### Incorporation of questions into lecture slides

The KEEpad system (KS) (KEEpad ltd, London, UK) was chosen as the audience response hardware and appropriate questions were integrated into eight (from a total of 14) key, 1 hour, pharmacology lectures, representing a mix of both basic and clinical pharmacology through PowerPoint using TurningPoint software (TurningPoint 2008). TurningPoint software is integrated into Microsoft PowerPoint and allows the inclusion of a range of question types into a lecture, for example multiple choice and true/false style questions [11]. Lectures were developed by the authors and questions were determined, and incorporated into the PowerPoint slides using TurningPoint, by the authors, prior to the sessions. Questions were based on the learning outcomes for each session. Questions, in the form of either a true/false or a multiple choice question, were incorporated throughout the lecture and appeared on the PowerPoint slides, prompting the students to answer, usually a defined time (30 seconds) was provided to answer the question and this was highlighted as a timer on the slide. As soon as all students had answered, or the time was up, the lecturer progressed the slides to display the correct answer and a graph outlining the responses of the students as a percentage per response. Questions at the beginning of the lecture generally assessed concepts covered in previous sessions thus allowing for repetition of threshold concepts and integration of concepts from more than one session. For the first lecture these questions were used to generate baseline information regarding the level of prior pharmacology knowledge of the student group. Questions which were incorporated in the body, or at the end, of the lecture assessed students understanding of concepts covered within that specific session.

### Formative Feedback from KS

**Figure 1:**
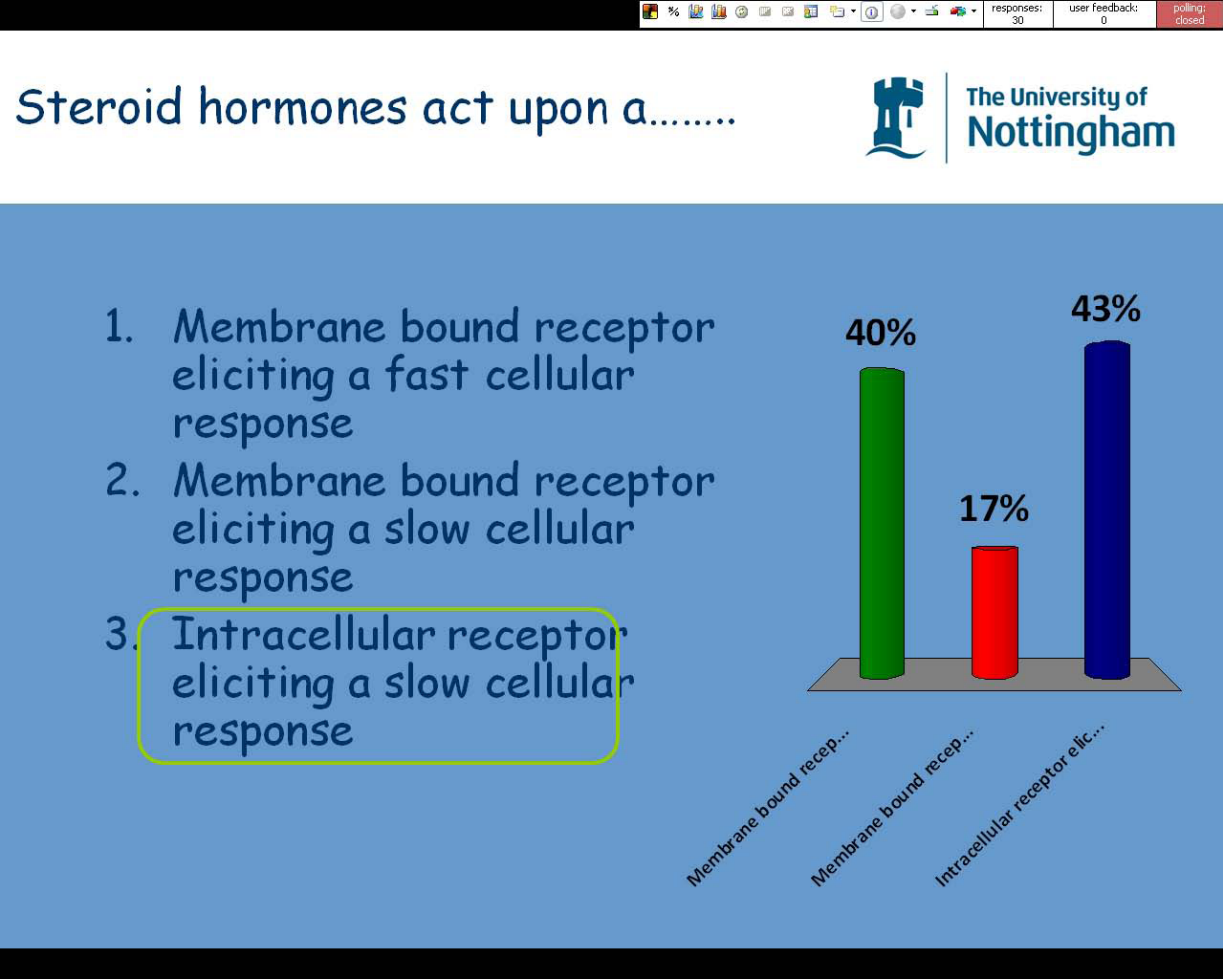
**Example screen shot of the KS (automated responses, not actual classroom data)**.

Students were randomly allocated a KS handset at the beginning of each session thus allowing for complete anonymity in relation to the results. The number of students who had answered the question was recorded by the KS, similarly a 30 second countdown timer was also incorporated into the question to encourage students to make a decision and answer the question. At the end of the countdown, the percentage of students opting for each available answer was displayed graphically and the correct answer was identified on the slide (Figure 1).

### Student perceptions of KS

On the final day of the module, students were invited to evaluate the use of the KS using questions incorporated into the audience response system and three paper-based open text box questions.

The evaluation was designed following discussion between the authors based on a paper-based questionnaire used when piloting the KS in a single lecture with a previous cohort of students. No issues with regard to either content or face validity arose.

Students were initially asked two yes/no questions in relation to enjoyment of the system and whether they had answered all the questions. A further nine, 5-point Likert scale questions (from strongly agree through to strongly disagree), were incorporated to allow students to express their opinion in relation to the usefulness of the KS in helping them identify their learning needs, maintain focus in the lecture, stimulate interest, and promote both understanding and integration of concepts. Students were also asked whether they felt the KS helped teaching staff track student understanding and whether they felt this system would be useful in other lectures (both pharmacology and non-pharmacology). The nature of the methodology used to collect the data ensured that the results of the survey were completely anonymous. A paper based questionnaire was also provided to allow students to comment in three open text boxes in relation to the following statements "if you did not enjoy using the Keepad system, please give reason"; "if you did not answer all the questions using the Keepad system please give reason" and "any other comments".

Numerical data from Turning Point was entered into SPSS (Version 14.0) and results were analysed using a Mann Whitney test.

### Focus group

#### • Selection of participants

The final question of the KS on the final day was "I would be interested in taking part in a focus group about keepads". Students who agreed to take part in the focus group were asked to complete a contact slip and place it in a plain envelope. All envelopes were placed in a box and a member of staff unrelated to the course was asked to randomly select 6 envelopes from the box. These students were then contacted with details of the focus group. Five of the six students invited to participate attended the focus group. The remaining student was unable to attend due to ill health.

#### • Facilitation of focus group

The focus group was conducted in a private room at the University over a lunchtime period with refreshments being provided. The focus group was conducted by an independent research assistant who was not known to any of the students and lasted a period of 90 minutes. Also present at the focus group was a second research assistant who sat on the outside of the group and made observational notes in relation to the body language and interactions between individuals within the group.

The discussion was digitally recorded using an MP3 recording kit and the recording was transcribed verbatim by a third research assistant.

#### • Analysis of data

The transcript was analysed independently by two members of the research team using a framework analysis technique [30,31]. Briefly, both researchers initially read through the transcripts of the focus groups then read through again highlighting, cutting and pasting sections which contained one or more discrete themes. Further re-reading and grouping of the identified themes into "key" themes or categories reduced the number of themes and highlighted overarching "super-themes" under which sub-themes were clustered [30]. The two researchers met to discuss the key themes which had emerged in their reading of the transcript, very little disparity was observed between researchers.

## Results

### Student Demographics

**Figure 2:**
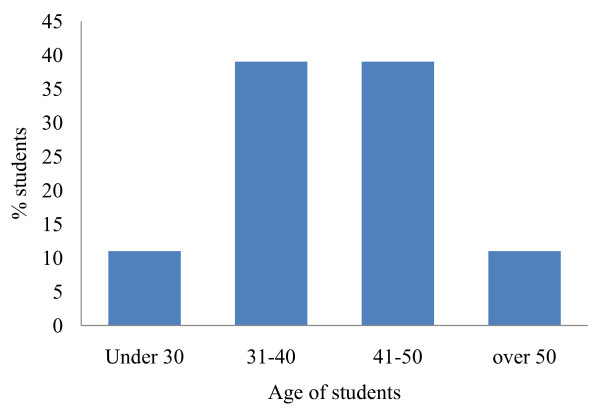
**Age range of students attending the non-medical prescribing course**.

All students attending the NMP course at this particular time were nurses with 14% being registered mental health nurses and the remaining 86% being adult nurses (nurses involved in the care of individuals over 18 years of age). A minority of the cohort (7%) were male with the majority (93%) being female. The age of the student population ranged from 27 to 56 years of age with 89% of the students being over 30 years of age and 11% being aged over 50 (Figure 2). Students had obtained their initial nursing qualification between 7 and 36 years prior to undertaking the NMP course.

### Student performance in relation to KS questions

A total of 127 questions were "asked" by the KS during eight lectures throughout the module, these could be grouped into 12 broad topic areas; links to clinical practice, general pharmacology, key pharmacological terms, absorption, distribution, metabolism, excretion, autonomic nervous system (ANS), endocrine, contraceptive, cardiovascular system and haemostasis. An average of 14.1 ± 0.5 questions were asked per lecture with 10 questions repeated in two or more lectures. For any given question, no fewer than 81.5% of students attempted an answer and no individual student abstained from answering all questions in a session. Analysis of the ten questions which were repeated in two or more lectures is shown in Table 1. There was an increase in the percentage of students who were correct in 9 out of the 10 questions and this was statistically significant in 3 out of the 9 questions. In the remaining question analysis of student responses showed a statistically significant decrease in the percentage of students who gave the correct answer.

**Table 1:** Student performance on repeated questions

	% Correct			Statistical significance	
		
Question	Time 1	Time 2	Time 3		
Agonists have affinity but no efficacy	87.5%	92%	88.9%	NS	

Insulin is a protein	59.3%	96%		< 0.001	Positive

Insulin is an agonist	96.2%	100%		NS	

Insulin promotes the storage of glucose	52%	96%		< 0.001	Positive

Irreversible agonists use which type of bonding	95.5	88.46		NS	

Oxidation, reduction and conjugation are all phase I metabolism reactions	44%	60%	76%	< 0.05	Positive

Pancreatic β cells produce which hormone	87.5%	100%		NS	

The binding between aspirin and COX is	91.7%	68%		< 0.05	Negative

The SNS acts to; increase heart rate increase bronchodilation increase GI motility	54.1%	62.9%		NS	

Which of these does not represent a drug target?	48%	68%		NS	

Pharmacology exam results from the cohort experiencing teaching with the KS were compared with the previous cohort who has not experienced KS teaching. Exam questions were grouped into 4 topics: basic concepts, kinetics, autonomic nervous system and endocrine, all of these topics had a KS component within the lectures. Students who had experienced KS teaching performed significantly better in the basic concepts (P < 0.001) and kinetics (P < 0.002) questions compared to the previous cohort (ANOVA with Bonferroni correction post hoc test). No statistical differences were observed between cohorts for the autonomic nervous system or endocrine system questions,

### Survey of student perceptions of the KS

Students were invited to quantitatively evaluate the KS as a teaching tool on the final day of the module. The response rate was (71%) and (78%) for the Likert questions and open questions respectively. 100% of students stated that they enjoyed using the KS and 92% stated that they answered all the questions.

In relation to students perceptions of the KS as a learning tool none of the students strongly disagreed with any of the statements. Indeed, 100% of students either agreed or strongly agreed that repetition of key concepts in different sessions was useful and that the KS promoted understanding of concepts. Students were similarly overwhelmingly positive about the usefulness of the KS in promoting identification of individual learning needs (92.0% agreement), maintaining focus in lectures (81.8% agreement), stimulating interest in lectures (83.3% agreement) and promoting integration of concepts (86.9% agreement). Furthermore 88% of students agreed that the KS allowed the lecturer to track student understanding. All of the students (100%) agreed that the KS would be useful in other pharmacology lectures and 75% agreed it would be useful in other parts of the curriculum (Table 2).

**Table 2:** Student feedback (%) on the use of the KS

	Strongly agree	agree	neutral	disagree
The KS helped me identify my learning needs	76.0	16.0	8.0	

The KS allowed the lecturer to track our understanding	60.0	28.0	8.0	4.0

The KS helped me maintain focus in lectures	50.0	31.8	9.1	9.1

Using the KS stimulated my interest in the lectures	50.0	33.3	16.7	

The KS was useful in promoting my understanding of concepts	54.2	45.8		

I found the repetition of key concepts in different sessions useful	91.3	8.7		

The KS was useful in promoting integration of concepts	39.1	47.8	8.7	4.4

I think the KS would be useful in other pharmacology lectures	95.8	4.2		

I think the KS would be useful in other parts of the NMP curriculum	45.8	29.2	16.7	8.3

KS, KeePad System.

Students completed an evaluation of the KS as a learning tool and were invited to make comments on 3 areas; "if you did not enjoy using the Keepad system, please give reason"; "if you did not answer all the questions using the Keepad system please give reason" and "any other comments". The qualitative feedback was overwhelmingly positive with 61 positive feedback and 9 negative statements received. No students commented in relation to not enjoying using the KS system. Only 2 students provided feedback on why they did not answer all questions, both stated that they ran out of time when they were unsure of an answer. The feedback was grouped into emerging themes which are outlined in Table 3. Clearly the students valued the opportunity to identify any areas of weakness and address these with extra learning, a typical comment was "*Really good way of monitoring how much I was learning and what areas I needed to work on*". Anonymity was an area which was valued by students as highlighted by this student "*as it was anonymous I think more students likely to have a go at answering questions - not intimidating if you had a go and got it wrong*".

**Table 3:** Themes which emerged from the qualitative feedback comments

Theme	Occurrence
Identified own learning needs	17

Anonymity	7

Exam preparation	7

General positive comment	7

Revising previous sessions	4

Wanted more	4

Clarity	3

Tailored teaching to group	3

Revision tool	3

Anxiety reassurance	3

### Focus group analysis

The focus group provided richer detail on the themes described in table 3, along with several themes which had not previously been raised by the students (Table 4). The overarching themes which emerged were that of enhancing student learning and enabling teaching. Student views appeared extremely positive with students using language which expressed a real intensity of feeling *'I thought they were absolutely brilliant'*.

**Table 4:** Themes which emerged from the focus group transcript analysis

Enhancing Learning	Improved focus and concentration	All students in the focus group agreed that using the KeePad system helped their concentration
	
	Tracking individual learning needs	All students expressed the view that the KeePad system had allowed them to track their own learning needs. Some intensity to the comments around this area 'really good (s6)'
	
	Improving confidence	4 out of 5 students commented around this area
	
	Exam familiarity	High frequency for 1 student but with agreement from a second student.
	
	Opportunity for Reflection	Students commented on the opportunity to consolidate what had been learnt in the current and previous lectures.
	
	Tutor Feedback	The benefits of both positive and negative feedback via the KeePad system were noted.
	
	Integration with other IT tools	1 student noted that the feedback from the KeePad system could be followed up by listening to podcasts.
Enabling Teaching	Clarification of concepts	3 of the 5 students raised this aspect of the Keepad system as a benefit. Raised on 3 different occasions.
	
	Improving teaching	3 of the 5 students raised this aspect of the Keepad system as a benefit. Raised on more than one occasion.
	
	Use in other areas	All students agreed the Keepad system could be useful in other areas of the non-medical prescribing curriculum although there was debate around how it would be used.

#### • Enhancing Learning

Within this theme, all students expressed positive views in relation to both the use of the KS improving focus and concentration within the lectures;

*'It made you focus on the questions that you were being asked and also on the lecture'*.

Similarly all students expressed the view that the KS had allowed them to track their own learning needs referring to this on more than one occasion;

*'I felt comfortable with the KeePads .... It did not matter whether you got it right or wrong, but you did learn from it, so if you got a question wrong you thought oh OK, well I need to look that up and make a little note' *(S5)

*'It is a good way of getting feedback on your strengths and weaknesses and understanding what you need to go and look at' *(S3)

*'It does allow you to assess where your weaknesses are without making you feel like a complete idiot in front of the whole class or tutors or whatever' *(S1)

Four of the five students commented in relation to the use of the KS improving their confidence in relation to developing pharmacology understanding;

"*It's not only nice to know not only what you are bad at but yes actually I can do this because that gives you the confidence*".

For one of the focus group participants it was the improved familiarity with exam style questions which was an important aspect of the KS;

*'I thought what was useful was it showed you how they structured the questions in the exam'*.

The usefulness of the KS in helping reinforce concepts was discussed by focus group participants in relation to both consolidation of their own learning; *'Well it just gives you time to assimilate the information. You know you are just trying to consolidate what you have learnt in the previous 25 minutes' *(S4)

and in terms of the continued feedback from lecturers

'And even if we got it right she would say and yes that is right because, sort of to reinforce it'

A further issue raised by the participants was the inter-relationship between the KS and the other forms of learning support available with one student remarking;

'*I just wrote down what (question) I did not understand, agonists for example. Then go home and look at the podcasts at home and say oh yes I know where I went wrong there'*

#### • Enabling Teaching

The second overarching theme identified by all students was that of enabling teaching with students discussing how use of the KS might improve teaching quality and increase lecturer satisfaction. One positive issue raised by three of the five students was the use of the KS to allow lecturers to clarify concepts which students might be struggling with;

*'But also if the group as a whole was poor in a particular topic it was seen that they were and the tutors then went over that little bit again, to try and explain, so it was just a way of enhancing the learning process' *(S5)

*'You have probably not got your point across clearly have you, if you have a lot of people in the group who have missed that point, you have not delivered it clearly so it gives them a chance to reiterate that point' *(S4)

*'And they could gauge how well we were doing, and if a lot of people got it wrong they didn't just re-explain it, they would re-word what they were saying and try and explain it in a different way.' *(S3)

The impact of the use of this technology on lecturer satisfaction was an unexpected outcome of the focus group discussion;

'I think it allows the tutor to reflect on whether or not they have pitched that information in a way that a majority of people have understood. Because that is the most important thing isn't it, if you have a lecturer who explains the same concept to you three times in exactly the same way you are no more likely to get it on the third time than you were on the first I don't think. So I think it is invaluable really'

"*they [the lecturers] will think oh actually yes they got that and that must be quite encouraging for them I think. It probably enhances their job satisfaction a bit*". The use of the KS in relation to other pharmacology teaching on the course and the potential benefits of this technology on improving the quality of teaching and allowing these lecturers to gauge this themselves was also an area highlighted by 3 of the 5 participants.

'I think with (lecturers name) he explained it and when we didn't understand he explained it again exactly the same and we didn't understand it so he explained it again and he didn't think how can I change this because they are not getting it. What different terms can I put it in to make it easier for them. He just said the same thing again and again, it's like no, and again and it was exactly the same'

Students were also positive about the use of the KS in other areas of the NMP curriculum including law, accountability, ethics and evidence-based practice.

'I think it would be quite useful in accountability and ethics. Just to focus on that particular area that.....and the understanding of the whole thing would come across if you were using the keepad'

Students were also able to see the wider use of the KS as a tool to generate discussion of subject areas which are more discursive and less factual in nature.

'Because we could all answer different things (in terms of ethics) and then we could use it as a discussion point'

## Discussion

Data shown here clearly demonstrates that students enjoyed using the KS and felt it helped promote understanding and integration of pharmacological understanding, this perception is backed up with evidence from the pharmacology exam demonstrating improved performance for the basic concepts and kinetics components of the assessment. Data from the KS itself demonstrated an improvement in student knowledge in relation to the majority of threshold concepts following repeated questioning and the reinforcement of concepts provided by the tutor feedback following each question.

The outcomes from the project were not only positive, but supported the pedagogical expectations discussed in the introduction, particularly that of identifying own learning needs and anonymity. The ability of the KS to provide students with an opportunity to reflect upon their own learning early in the module, through prompt formative feedback, is highly beneficial - both to the student and also to the lecturers [14,25-27]. Students benefit by identifying areas of weakness and obtain an early opportunity to revise these problem areas thereby reducing stress and anxiety later in the course which could impact upon exam success. Indeed, reduction in anxiety, use as a revision tool and preparation for examination were themes which were highly cited by the students (Table 3). The use of the KS not only acted to engage students in the pharmacology teaching thus promoting enthusiasm and understanding but also acted to develop student confidence in their own ability and capability thus acting as an empowering exercise, these findings are in agreement with Graham et al who describe the ability of ART to empower or compel reluctant learners to engage in teaching [32] and Slain et al who describe the development of active learning in a group of healthcare (doctor of pharmacy) students [33]. This engagement and empowerment is particularly critical for our group of students, many of whom do not have a traditional educational background and lack confidence in their biological science knowledge.

The KS was also beneficial for the lecturers who were able to identify areas or concepts which were problematic - for example, when the question "oxidation, reduction and conjugation are all phase I metabolism reactions" was asked initially, the correct response was only 44% - this signalled to the lecturer that students had not grasped this concept fully and the lecturer was able to revisit the material in the next lecture and also ask if another lecturer could integrate the question into a clinical lecture later in the module. Subsequent "asking" of the same question achieved a correct response of 60% and 76% - a significant improvement. This does not just allow students to memorise the material, but to integrate concepts across several lectures, therefore several different pharmacological areas. As demonstrated in Table 1, there was one question in which the response rate decreased with subsequent lectures, we hypothesise that this is due to the concept being taught across 2 sessions and by 2 lecturers (one of whom was an outside speaker) who may have used different language to describe the concept. Identification of this as a potential problem is also important information for the lecturing staff allowing them to clarify the language used and ensure consistency across all teaching areas.

The improvement observed in student performance in the basic concepts and kinetics components of the exam, but no statistical improvement in the autonomic nervous system and endocrine system questions was interesting to the authors. The autonomic nervous system and endocrine system teaching covers relatively clinical areas which many of the students would be aware of from their day to day work as nurses, the basic concepts and kinetics teaching would, on the whole, be a completely novel area of learning. We propose that the students feel more confident answering questions on the clinical areas of teaching and that the KS had particular impact on learning where the topics were novel. Other investigators have assessed the impact of ART on student recall; Schackow et al assessed the recall of a group of post-graduate medical trainees immediately post-teaching and 1 month later. Students who had received lectures combined with ART had significantly higher results than those who had received traditional lectures [34]. Slain et al have published similar findings on the positive impact of ART on Doctor of Pharmacy students; students who had received teaching incorporating ART achieved significantly higher grades than students who had not received this style of teaching in two out of three assessed courses [33]. Interestingly, Gauci et al suggests that low achieving students may gain most from the use of ART [35]; this is an aspect that we have not yet investigated. Although the findings of improved exam outcome are positive, one must be aware of the limitations and not draw too strong a conclusion; the effect may simply be a cohort effect and our findings are limited to a single group of students at one UK University.

Students could also perceive the potentially beneficial impact on lecturers of including the KS in their lectures. Focus group participants were particularly vocal regarding the benefits of the KS in helping lecturers to focus on the key learning objectives, for example "*if they had to incorporate the KeePad into that [the lecture] it might make them focus on actually they need to know this*". Although this was an area that the authors were, as the main lecturers on the course, aware of, we were surprised that the students felt so strongly about this outcome of the KS.

While the results shown here relate specifically to the development of pharmacology understanding there is considerable potential for the use of the KS in other 'factual' areas of the curriculum including areas such as the legal aspects of prescribing and the basic principles of evidence-based practise. Moreover in this group of students there is the potential to utilise the KS to generate discussion in areas such as accountability and ethics.

While the KS allowed staff to determine what percentage of students struggled with basic concepts, the anonymity of the system did not allow identification of these specific students leaving the individual student to determine their own learning needs and how to address these. This could prove to be a difficult task for some students and it may be that the use of identified individual KeePads would allow lecturing staff to intervene with supportive measures at an earlier stage in the course allowing the development of fundamental concepts which can then be used to scaffold other knowledge.

One of the limitations of this study is that it is concerned with a very specific group of students from non-traditional educational backgrounds at a single institution and as such the results may not be replicated in other groups of students.

## Conclusions

Further improvements to the use of the KS will be introduced with future cohorts, several students indicated that they would like all pharmacology lectures to contain Keepad questions, this is an area for the teaching team to develop in collaboration with external speakers, in particular those who teach concepts which are integrated throughout several lectures. One way in which we could improve the feedback to students is provide students with an identified KEEpad, this would allow the lecturer to review the responses and identify students who were struggling with any key, or threshold concepts.

In conclusion, use of the KS to provide instant formative feedback has had a positive impact on student learning in the non-medical prescribing module. In particular, students highly rated the opportunity to identify learning needs early in the course, revise material and prepare for the summative examination. Audience response technology is a pedagogically sound way to enhance student learning across several learning styles and educational backgrounds with a low impact on lecturer time and resources.

## Competing interests

The authors declare that they have no competing interests.

## Authors' contributions

AM and JSL conceived of, designed the study, obtained the funding and analysed the data. Both AM and JSL drafted the manuscript. All authors have read and approved the final manuscript.

## Pre-publication history

The pre-publication history for this paper can be accessed here:

http://www.biomedcentral.com/1472-6920/10/73/prepub
